# Queer Theory and Biomedical Practice: The Biomedicalization of Sexuality/The Cultural Politics of Biomedicine

**DOI:** 10.1007/s10912-018-9526-0

**Published:** 2018-08-03

**Authors:** William J. Spurlin

**Affiliations:** Department of Arts and Humanities, Brunel University London, Uxbridge, England UB8 3PH UK

**Keywords:** Biomedical knowledge, Queer theory, Sexual health, Homosexuality, Gender identity disorder, Gender dysphoria, HIV/AIDS

## Abstract

This article works across multiple disciplinary boundaries, especially queer theory, to examine critically the controversial, and often socially controlling, role of biomedical knowledge and interventions in the realm of human sexuality. It will attempt to situate scientific/medical discourses on sexuality historically, socially, and culturally in order to expose the ways in which “proper” sexual health in medical research and clinical practice has been conflated with prevailing social norms at particular historical junctures in the 20th and 21st centuries. How might the relationship between clinical and cultural spheres be better engaged in biomedical knowledge and clinical practice in understanding sexual health, given the impact of homophobic and transphobic assumptions in the diagnostic histories of homosexuality and Gender Identity Disorder in Childhood, a new diagnostic category introduced into the *DSM* following the removal of homosexuality from the *DSM-III*? The article will argue further that biomedical knowledge is always already mediated through culture by analyzing normative racial, gender, class, and sexual ideologies that regulated early understandings of the epidemiology of the HIV/AIDS pandemic in the West and in the postcolonial world while informing global health policy on HIV/AIDS. The article concludes by examining the implications of medical education for both LGBTQI patients and medical professionals, for understanding gender and sexual rights as human rights, and for thinking about new kinds of interventions, contestations, and struggles to resist continued homophobic and transphobic assumptions in biomedical practice today and their ongoing effects in the everyday world.

One of the most politically contested axes of social subjectivity in contemporary culture is that of sexuality. While analyses of sexuality in biomedicine and in the humanities have, for the most part, developed along separate trajectories for different purposes, this article works across multiple disciplinary boundaries such as medical history, global and social health policy, postcolonial studies, and queer studies, as a way of examining critically the controversial, and often socially controlling, role of biomedical knowledge and interventions in the realm of human sexuality. In his book *The Birth of the Clinic*, Michel Foucault speaks of a significant discursive shift in biomedical knowledge around the end of the eighteenth century centered around “a new ‘carving up’ of things and the principle of their verbalization” through the process of the medical commentary (1973, xviii), which implied a new relationship between the *perceptible* and the *stateable*, between what is seen and what is said, thereby instantiating a redistribution of the relation between signifier and signified, that is, “between the symptoms that signify and the disease that is signified” (xviii-xix). In other words, according to Foucault, the medical commentary assumes an excess of the signified over the signifier, a residue of thought that has not yet been articulated in language so that the act of commenting is thought to give voice to that which has not yet been explicitly stated and is thus allowed to speak—that is, it uncovers deeper meanings by stating what has been said while simultaneously (re)stating what has not been specifically said, but has been signified (xvi). The discursive shift from the totality of the visible to the overall structure of the expressible is what occurs in the medical commentary; as Foucault more succinctly argues, “the clinical gaze has the paradoxical ability to *hear a language* as soon as it *perceives a spectacle*” (108). The mediation of the medical commentary is thought to strengthen the bond, suture the gap, between signifier and signified, between a symptom and its meaning, and give biomedicine a new discursive structure and a veneer of scientific precision, but the clinical gaze of which Foucault speaks is not without its concomitant social and cultural interpretations of the symptoms and signs of illness and disease.

Not only was there a shift in the discursive structures of western biomedicine at the end of the eighteenth century, there was a simultaneous epistemological shift in biomedical practices to the extent that medicine became linked to the destinies of nation-states. According to Foucault, this meant that medicine was no longer confined to a body of knowledge and techniques for curing ills nor concerned with the qualities of vigor, suppleness, and fluidity that were lost in illness which medical practice could restore, but biomedicine assumed a normative posture and became authorized to dictate the standards for the physical and moral relations of the individual and the broader social world in which he or she lived. As Foucault theorizes, since the nineteenth century, medicine “was regulated more in accordance with normality than with health”; that is to say, in considering the life of groups or societies, the life of the race, as well as psychic life later in the century, biomedical concepts became structured around the polarity of the normal and the pathological (34-35). Interestingly, it is also at the end of the eighteenth century, according to George Mosse, that the rise and proliferation of modern nationalisms in Europe were linked to middle class norms of the body and sexuality (Parker, Russo et al. 1992, 2), thus instantiating a link between the moral and physical (and later psychological) health of the individual and the health of the state—and the conflation of good health with social conformity.

The legacy of these two historical shifts articulated by Foucault, as well as the fusion of the medical and juridical spheres, which often forms the basis for citizenship, rights, and national belonging, are especially important since biomedical knowledge, and its concomitant social authority on sexuality, not without its political biases, had, from the nineteenth century, not only influenced and buttressed colonial power and rising fascist movements in Europe in the early twentieth century, but also, in the aftermath of imperialism, formed the basis for directing social health policies within western contexts and in the postcolonial and developing world. Coincidentally, it is within the context of biomedical and psychiatric discourses in the mid-nineteenth century that homosexuality, as a form of sexual identity, appears, as Foucault elaborates in his *History of Sexuality*.^1^ But it is important to note that these significant shifts in biomedical discourse and practice around disease and the frequent invocation and citation of biomedical science in juridical practice enabled the biomedical articulation of homosexuality as such. Further, as Dagmar Herzog articulates in *Sexuality in Europe: A Twentieth-Century History*, sexuality was burdened with enormous significance over the course of the twentieth century and acquired growing political salience, given the separation of sexuality from reproduction, which became apparent not only through the rising availability of birth control but also through heightened expectations of pleasure, particularly for women, and a general preoccupation with sexual orientation, sexual rights, and sexual norms (2011, 2-3). In this article, I am interested in interrogating the relationship between scientific opinion as informed by/informing social norms alongside the history of sexual health at particular junctures in the twentieth and twenty-first centuries and the broader politics of gender and sexuality that surround biomedical knowledge.

Given that queer theory functions both as a mode of analysis and as a strategy of opposition that critiques normativities imbricated within a wide range of social categories and social institutions, including, but not limited to the body, gender, healthcare, reproductive politics, the family, and citizenship, in addition to, and alongside, sexuality, my work is shaped by a Foucauldian analysis in situating biomedical discourses on sexuality historically, socially, and culturally in order to analyze their rhetorical appeals to scientific truth and rigor. My analysis here attempts to expose the ways in which gender and sexual health, in biomedical research and practice, is shaped by and shapes prevailing social norms enabled by the historical shifts I have mentioned earlier. Using queer theory as an analytic lens, I would like to explore the implications, contradictions, and collusions at work in discourses surrounding the diagnostic histories and clinical practices around homosexuality that followed the post-war years, the later diagnostic category “Gender Identity Disorder in Childhood” or GIDC, which replaced homosexuality following its removal from the *DSM-III* in 1980, the more recent diagnostic category “Gender Dysphoria in Children” in the current *DSM-V* (American Psychiatric Association 2013), the identification of risk groups for sexually transmitted infections (STIs) such as HIV/AIDS, and the broader politics of gender and sexuality that surround biomedical knowledge.

Nowhere in the clinical literature is the politics of diagnosis more evident than in the history of the listing of homosexuality in the *Diagnostic and Statistical Manual of Mental Disorders* (*DSM*) by the American Psychiatric Association (APA) in the period following the publication of the Kinsey report in 1948 through the historic 1973 decision to delete homosexuality as a diagnostic category. The first edition of the *DSM*, published in 1952, listed homosexuality as psychopathological and as a sociopathic personality disturbance during a time of intense social conformity in the Cold War era. Since the Kinsey report shattered the myth of the effeminate male homosexual and indicated that men with homosexual histories could be found in every age group, social level, occupation, and geographical area (1948, 627), this raised the possibility that gay men could escape detection, and, as Robert Corber argues, linked them with Communists who could conspire to overthrow the US government and subvert its national institutions from within. A resistance to the domestication of gender roles, according to Corber, also raised suspicion toward those men who refused to settle down, raise a family, and take on the roles of breadwinners and homeowners (1997, 11-12). With the rise of social activism in the 1960s against the conflation of homosexuality with mental illness, the *DSM-II*, published in 1968, considered homosexuality as indicative of psychopathology but removed it from the category of sociopathic disturbance, listing it instead under such other “sexual deviations,” as fetishism, pedophilia, transvestism, and exhibitionism.^2^ Clinical work from this period, especially the ten-year study of the etiology of male homosexuality by Irving Bieber, deliberately shifted psychoanalytic attention away from the role of constitutional factors in the development of homosexuality, which Freud quite adamantly indicated as important to consider,^3^ to oedipal and pre-oedipal experiences. The clinical research of this era also promulgated the all-too-familiar stereotype that a high proportion of gay men had “close-binding” mothers who sexually stimulated their sons through over-close intimacy and seductiveness, showed undue concern for their sons’ health and safety, and interfered with the relationship of their sons to their fathers and peers, both of whom enable the process of masculine identification through maternal separation (Bieber et al. 1988, 79-81). At the same time, Bieber’s study found patterns of prehomosexual childhood as a distinguishing factor of the one hundred six homosexual men studied, compared with the childhoods of one hundred heterosexual men in the control group. Seventy-five percent of men in the homosexual group reported excessive fear of injury in their childhoods, girls as primary playmates in a third of the group, and participation in the “usual” games of boys in less than one-fifth (204). As adults, the homosexual men studied exhibited such behaviors as exaggerated shrugging, “wrist-breaking,” lisping, hand-to-hip posturing, and effusiveness, and Bieber reports that these patterns of feminine behavior in males is less an emulation of femininity than a caricature of it, since such behavior in females would appear “bizarre” rather than feminine (188-89). This predates (but ironically addresses) Judith Butler’s much later theory of gender as performative and constituted by “the political and cultural intersections in which it is invariably produced and maintained” (1999, 6) through the citation and embodiment of gender norms.

Following intense activism within the medical profession and outside, the American Psychiatric Association set up a committee to review the diagnostic status of homosexuality in the early 1970s, and on the basis of the committee’s recommendation, the APA decided to delete homosexuality from the *DSM* in 1973. Rather than signifying the last reference to homosexuality in the official nomenclature of biomedicine, the *DSM-III*, published in 1980, did not contain an entry for homosexuality (except for ego-dystonic homosexuality which was deleted in 1987 in *DSM-III-R*) but added a new diagnostic category “Gender Identity Disorder in Childhood” or GIDC. GIDC, which claimed to align biological sex with the notion of a core gender identity or CGI (see Stoller 1964), has been enforced therapeutically on gender-atypical children, especially boys, who are least capable of resisting it, and treatment for GIDC was often based on the fear of eventual gay outcome. Moreover, the so-called symptoms of GIDC were not remarkably different from those described in earlier research on the etiology of homosexuality in the 1950s and 1960s which served as the intertext for symptomatic descriptions of GIDC in the *DSM-III* (1980) and the *DSM-IV* (APA 1994). Demonstrative of this questionable professed shift from homosexuality to GIDC, Richard Friedman, who chaired the APA Committee which recommended the removal of homosexuality as a diagnostic category from the *DSM*, supported GIDC in his book *Male Homosexuality: A Contemporary Psychoanalytic Perspective* wherein he spoke of gender-atypical boys as having “female-like symptoms” (1988, 199) and argued that most childhood effeminacy results in homosexuality and that most adult homosexuality is preceded by some kind of prepubertal *gender disturbance* (212).

Additionally, as with the earlier research on homosexuality, Susan Coates and Kenneth Zucker, internationally-known experts on GIDC, have described mothers of feminine boys as overbearing and pathogenic through transferring their unresolved trauma on to their sons. For Coates and Person, for example, this suggests a “disturbed” mother-child interaction and that boyhood femininity is symptomatic of separation anxiety and a desire “to restore a fantasy tie to the physically or emotionally absent mother” (708). As with the earlier work on homosexuality, mothers are blamed for the gender nonconformity of their sons. In the years leading up to the publications of the *DSM-V*, LGBTQ activists opposed vehemently Kenneth Zucker’s appointment by the APA in 2008 to chair the workgroup on Gender and Sexual Identity Disorders for the next edition of the *DSM* largely because of his work on gender identity disorder in children. In 2015, Zucker’s Gender Identity Disorder Clinic, part of the Toronto Centre for Addiction and Mental Health (CAMH), was closed. Following an external review critical of the way the clinic treated children and young patients struggling with issues related to their gender identity, Zucker’s dismissal, according to the Toronto *Globe and Mail*, was a result of his work no longer being “in step with the latest thinking” given that it suggested that gender nonconforming children be discouraged from becoming transgender adults, which many in the transgender community viewed as a form of conversion therapy (Anderseen 2016).

The current *DSM-V* replaces the diagnostic category GIDC with “Gender Dysphoria” with a separate section dedicated to children, signifying the further displacement of the diagnostic category rather than its disappearance. It describes gender dysphoria as a marked incongruence between one’s experienced/expressed gender and one’s assigned gender, the latter of which is based on, and conflated with, natal sex. The diagnostic criteria include a child’s strong preference for the clothing of the other gender; a strong preference for cross-gender roles in make-believe or fantasy play and for the toys, games, and pastimes more typical for the other gender; and the attendant stress that accompanies such incongruence (APA 2013). While the *DSM* now stipulates that the term “gender dysphoria” is more descriptive than the previous usage of “gender identity disorder” as it related to children in the previous editions (APA 2013), the diagnostic criteria for gender dysphoria in children, and the attendant descriptors, seem remarkably similar to those for GIDC, and still echo, to some degree, earlier work on the etiology of homosexuality with regard to its tropes in describing gender variance in children.^4^

What the diagnostic history of homosexuality, GIDC, and gender dysphoria in children point to is to the ways in which biomedical knowledge is structured around the polarity of the normal and the pathological to the extent that all have served to maintain heteronormative gender norms. This discursive formation supports Judith Butler’s claim that sexuality in culture “is regulated through the policing and the shaming of gender” (1993, 238) while providing a powerful and legitimate discursive and clinical apparatus for that very shaming and policing and its medical and social reinforcement. Moreover, these instantiations of homophobia, transphobia, and misogyny in the clinical literature, past and ongoing, have not only been condoned biomedically and clinically but continue to provoke social condemnation, discrimination, the incitement to violence, and the bullying of children who cross-gender identify, with higher rates of suicide among them, in addition to various form of social exclusion, actual or imagined, against gender nonconforming children and gender and sexual dissidents more broadly, while continuing to undermine erotic autonomy and gender expression as fundamental human rights. In addition, mothers have been pathologized in clinical literature as overprotective, indulgent, seductive, overanxious, or unhappily married, and not the slightest consideration has been given to the possibility for mother’s and son’s subjectivities affording greater closeness and empathy (Corbett 1999, 129). Moreover, to what extent will a clinical diagnosis of gender dysphoria in a child be related to a fear of possible gay outcome by therapists, medical professionals, and parents? As I have written previously, what also needs to be pointed out is that transgender identification in children points to the failure of the matrix of heterosexuality to legislate itself fully. In these post-theoretical, post-queer times, how can the trans rupture in the matrix of gender intelligibility be welcomed as producing new identificatory sites and new conceptual apparatuses for understanding the psychological growth of children who cross-gender identify (who may or may not turn out to be gay) and LGBTQ people (who may or may not conform to prescribed gender norms) (Spurlin 1998, 91)?

Another site that encapsulates the regulation of biomedicine through the vicissitudes of social normativities lies in the history of the HIV/AIDS pandemic. Early manifestations of AIDS-related illnesses exposed a gap in biomedical knowledge and thinking in the early 1980s when gay men were identified as a primary risk group and as primary carriers of the virus, without a clear understanding of the epidemiological foundations of HIV/AIDS. Before the isolation of the human immunodeficiency virus, and certainly after, biomedical science contributed to the emergence of new discourses of sexual perversion centered on metaphors of social defiance, erotic indulgence, hedonism, and moral laxity around the transmission of HIV either sexually or through shared needles used intravenously. Ironically, this was right at the same time that the *DSM-III* (the first edition of the *DSM* not to list homosexuality as mental disorder) was published, so that homosexuality became once again, but differentially, cast as a perversion. Those who were diagnosed with HIV seropositivity, as Susan Sontag notes, were cast as deserving of blame (1989, 26) to the extent that gay men, intravenous drug users, and those of Haitian descent were seen as “disposable” groups and the primary risk groups for acquiring HIV in the United States. As Cindy Patton observes, the pandemic gained its social meaning over time by building on already deeply seated social prejudices surrounding race, class, gender, sexuality, and addiction. Those who suffered the specter of decadence, decay, and death associated with HIV/AIDS, she argues, were seen initially as isolated cases, which helped to erase the social realities that shaped the growing epidemic (1990, 25). “Good health” in the early days of HIV/AIDS was defined by the medical profession, social health policy, and the media through such vectors as whiteness, western location, rationality, emotional self-control, and middle-class values such as hard work, productivity, and moderate habits as safeguards against licentiousness, sexual indulgence, and addictive behaviors that would lead to decadence and disease, not remarkably different from the codes of bourgeois morality that shaped rising nationalisms in late eighteenth-century Europe. This points not only to racial and class hierarchies in understanding HIV/AIDS in the early 1980s but also substantiates the fact that biomedical knowledge is always already mediated and produced through and around particular cultural symbols (Patton 1990, 67) and that the scientific authority it holds has been used politically as a form of sanctioned governance to incite and perpetuate discriminatory social practices.

In considering an important historical precedent, biomedical discourses on sexuality were closely linked to the racial politics of National Socialism, whereby Nazi doctors studied homosexuality as a form of social degeneracy and as a threat to racial hygiene, appealing to the authority of biomedical science in order to maintain rigid social distinctions between the genders and the procreative responsibility of Aryan citizens. In the mid-1930s, medical doctors in Germany argued nearly unanimously that homosexuality, medically speaking, was a threat to public health; Germany’s leading public health journal at the time *Der Öffentliche Gesundheitsdienst* described homosexuality as a psychopathology (Proctor 1988, 212),^5^ not that far removed from clinical descriptions of homosexuality in the *DSM-I* nearly twenty years later. Arguing against homosexuality as biologically determined, one Nazi doctor, in writing for the Reich Office of Racial Policy in 1938, proclaimed that homosexuals, like Jews, were state criminals and “not ‘poor, sick’ people to be treated, but enemies of the state to be eliminated” (Proctor 1988, 213).^6^ Going back further, Mosse and others have noted that late nineteenth-century medical literature in Europe, very much influenced by scientific racism at the time, often conflated the pathologies of male Jews and homosexuals—both were thought to be prone to hysteria, nervous bodily distortions, and feminine tone of voice and bodily movements (1999, 64).^7^

The biomedicalization of homosexuality under National Socialism was by no means a momentary aberration as nationalist discourses in much of the postcolonial world today read homosexuality as a colonial import and as a form of western decadence that is foreign to indigenous cultural traditions. Western biomedicine has played a role historically as a tool of imperial power. Frantz Fanon, an early postcolonial theorist originally from Martinique who studied medicine and psychiatry in France, and served a medical residency in Algeria and became involved in Algeria’s struggle for independence, noted that medical knowledge was one of the most insidious tools of colonial conquest and contributed to the dehumanizing logic of colonial rule (1963, 296). Similarly speaking of the French colonial conquest of Algeria, Richard Keller notes in *Colonial Madness* that physicians, surgeons, and pharmacists saw diagnosis and treatment as a contest over civilization alongside health and disease (2007, 11). In terms of sexuality, this meant that European physicians in the late nineteenth and early twentieth centuries read Africa in particular as “a space of savage violence and lurid sexuality” (1). Largely as a result of the effects of the so-called civilizing mission of colonialism, and the remnants of homophobic laws that often have their origins in colonial administration, HIV/AIDS sufferers in many postcolonial societies today bear the stigma of sexual deviance and moral laxity, and these markings have been shaped by a history of imperialism, outdated western psychiatric opinion on the etiology of homosexuality, and causal links between homosexuality and HIV/AIDS constructed by western biomedicine in the early history of the pandemic. Yet the effects of the biomedical justification of colonial rule continue in the contemporary surveillance and tracking of HIV/AIDS by global health institutions such as the World Health Organization (WHO) and UNAIDS. As Cindy Patton has argued, the term “African AIDS,” used early in the pandemic, mobilized racist ideologies of unchecked, unbridled sexuality amongst indigenous Africans and amongst blacks in general.^8^ The rhetorical strategies of medical thought-styles in representations of HIV/AIDS globally, Patton notes, have been deeply layered with social ideologies around race, class, and sexuality, and have the power “to structure the terms through which bodies become visible as the locations of disease, of an epidemic” (2002, 26).

Another problem with the effects of imperialism was the initial reluctance of many African nations to admit to a presence of homosexuality within their borders and even higher rates of HIV infection than were originally assumed or predicted. This was tied to deep-seated historical anxieties about discursive appropriations of African sexuality by the West in decadent terms, a legacy of colonialism which remains, as with the term “African AIDS,” in discourses surrounding the global surveillance and tracking of HIV/AIDS. At the same time, the reading of homosexuality as un-African by some strands of African cultural nationalism produced a significant gap for those at risk for HIV who escaped the categories of the West, given that some indigenous African men practiced anal sex with other men but did not identify as gay and lived heterosexual lives publicly, which was compounded by the fact that the WHO saw HIV transmission in Africa largely in heterosexual terms in the early days of the pandemic. AIDS educators were not initially sensitive to the fact that anal sex has different meanings and values in different cultural systems that needed to be addressed in helping those men, who engaged in the practice of anal sex with other men as partners, recognize that safer sex applied to them as well, even if they resisted taking on a gay identity as it is understood in the West. The adoption of the descriptive phrase “men who have sex with men,” or MSM, by the WHO’s Global Programme on AIDS provided a thinly veiled screen, or closet, at the time, not of mere secrecy but of a “safe” identity that was more legibly heterosexual but later, it was realized, no less at risk for HIV transmission or infection. The problem with western understandings of homosexuality, initially imposed by global health organizations on indigenous men who have sex with men, was not so much the conflation of anal sex with homosexuality but the conflation of sexual *practice* with sexual *identity*, which places Foucault’s proposition of a shift in homosexuality in the nineteenth century from a temporary aberration to an emergent identic category (1980, 42-43) even more firmly in the West. More important, such imperialist thinking missed significant forms of HIV transmission not immediately apparent to western thinking, which was based on the confluence of sexual practice with sexual identity and resulted in subsequent gaps and delays in education and prevention programs in large parts of sub-Sahara Africa early in the pandemic.

Additionally, placid assumptions in the West that the availability of anti-retroviral (ARV) medication no longer signifies eventual death for those who are HIV-positive fail to recognize that this is precisely what it does signify for the many indigenous Africans in sub-Sahara Africa dying from AIDS-related illnesses each day. South Africa has the highest prevalence of HIV/AIDS in the world, estimated by the South African government’s statistical report of 2015 to be at about 6.19 million of its total population of 54.96 million with the highest impact of HIV/AIDS falling on indigenous African women (Statistics South Africa 2015). A report on violence against women and HIV/AIDS by the UNAIDS Coalition on Women and AIDS and the WHO points to the everyday realities of gender inequality and intimate partner violence in South Africa. It is difficult for women, particularly younger women, to negotiate condom use with intimate male partners. High rates of gender-based violence and rape often serve as barriers to women seeking HIV testing, anti-retroviral treatment, and access to services which could prevent mother to child transmission (UNAIDS Global Coalition on Women and AIDS and WHO 2005). Alarming numbers of indigenous African women who identify as lesbian experience “corrective rape” as a cure for their so-called aberrant desires, placing them at risk for HIV/AIDS as well.

Another issue pointing to the high prevalence of HIV/AIDS in South Africa is that in the late 1990s and in the early part of the last decade, some global health officials argued that those living in poverty were not literate enough to follow the prescribed regimen of treatment for taking ARV medication; this racist argument, in turn, was appropriated by western pharmaceutical companies as a rationale for not lowering the cost of the drugs so that they would be affordable to poorer South Africans, arguing that a failure to take the drugs responsibly could lead to drug-resistant strains of HIV. The Treatment Action Campaign (TAC) in South Africa has been the most vocal and visible lobby fighting for the rights of HIV-positive people for equal access to treatment; in the late 1990s, TAC willfully ignored international trade agreements pertaining to the production, import, and use of less costly generic versions of patented ARV drugs for the treatment of HIV infection. More recently, TAC has put pressure on UNAIDS not to overstate the likelihood of ending HIV/AIDS given the deleterious effects this could have on donorship for global HIV/AIDS funding and the politics of sexual healthcare in the developing world. The French nongovernmental human rights organization, Médecins Sans Frontières/Doctors without Borders, has worked in some of the most impoverished townships in South Africa providing ARV and TB medication to those living with HIVAIDS who are facing the challenges of poverty, marginalization, and stigma. Their work defies earlier biomedical discourses on HIV/AIDS in Africa purporting that poor Africans were too uneducated to take the medications responsibly. Given South Africa’s history of disobedience, struggle, and resistance to oppressive regimes, this work calls attention to the production and distribution of power which certainly is imbricated with biomedical thinking around ARV access and pricing in the developing world.

In conclusion, if sexual desire can become a mechanism for various forms of social manipulation, how does western biomedicine continue to play a significant political role in the cultural management of gender and sexual norms? How might the relationship between the clinical and cultural spheres be better engaged in biomedical knowledge and practice, especially around the topic of sexual health, given biomedicine’s historic failure to recognize the influence of homophobia and transphobia in, and their reproduction through, the diagnostic histories of homosexuality and GIDC, and the racial, gender, class, and sexual ideologies that constructed early readings of the HIV/AIDS pandemic in the West and in the postcolonial world? While the identification of risk groups is key for understanding patterns of disease transmission, especially in the case of HIV/AIDS in the context of sexual health, and is essential to helping people to avoid becoming ill, what social and cultural ideologies are operating in epidemiological discourses about specific risk groups and their behavior? Where will this theorization occur?

What the diagnostic histories and medicalizations of homosexuality, gender identity disorder, gender dysphoria, and HIV/AIDS also indicate is a link between compulsory heterosexuality and compulsory able-bodiedness as theorized in contemporary disability studies. As Robert McRuer argues, medically speaking, the ideal heterosexual subject is one whose sexuality is not compromised by disability such as queerness; whereas the ideal or successful able-bodied subject is one whose ability is not compromised by queerness, that is, by disability (2010, 387). As evident from the earlier discussion, medical science historically has seen the queer body as a disabled or diseased body, and the remnants of colonial medicine that operated in the early days of the HIV/AIDS pandemic held the white, western, heterosexual body as the standard of normality and health. These links are important since biomedicine as a discipline, and as a form of knowledge production, addresses various forms of disability and disease but historically without a theorization of the cultural politics that dictate, in this context, the ways in which “compulsory heterosexuality is contingent on compulsory able-bodiedness and vice versa” (McRuer 2010, 384), and, more broadly, the social and cultural conditions that inform the normal/pathological split.^9^

The ongoing heteronormative slant in medical education in North America and elsewhere is certainly not promising; studies have shown that specific healthcare issues pertaining to lesbian, gay, bisexual, and transgendered patients are not being adequately addressed in clinical training, pointing to a cultural blind spot, another instantiation of the normal/pathological binary, and a refusal to engage with the exceptions and contingencies of what is prescribed as normative. What are the social repercussions for critical healing? A study by the Stanford University School of Medicine, published in September 2011 in *JAMA*, found that students at one-third of 176 responding medical schools in the United States and Canada received *no* gay-related healthcare education during their clinical years; only three in five schools provided instruction in eight or more of the sixteen health issues of concern to lesbian, gay, bisexual, or transgendered people, including sex reassignment surgery, inaccessibility to healthcare, safer sex, and chronic (sexual) disease.^10^ A more recent study on implicit bias against sexual minorities in biomedicine, published in *Academic Medicine* in 2015, found that 46% of heterosexual first-year medical students in the US expressed some explicit bias against lesbians and gay men and that 82% held implicit biases; that is, they held ingrained, but unrecognized or unconscious, beliefs toward the target group (Fallin-Bennett 2015, 549). The study recognizes that further work needs to show that biases such as these actually affect LGBT patient care, but other studies have shown that implicit racial bias, for example, does affect physician decision making, and that it is reasonable to assume that LGBT patients are also at risk for discrimination and compromised care in the biomedical context (549). A study published in *Virtual Mentor* the previous year by Jonathan Metzl and Dorothy E. Roberts found that extra-clinical stigma, socioeconomic factors, and cultural politics shape diagnostic and treatment disparities just as these similarly shape the material realities of patients’ lives. African-Americans were much more likely to receive diagnoses for schizophrenia as compared with white patients but less likely to be diagnosed with depression or bipolar disorders compared with their white counterparts (Metzl and Roberts 2014, 675). The article asks that the clinical situation be understood as constructed by political, economic, racial, and gendered social structures and hierarchies that produce vulnerability for particular groups of patients (682).

Yet, while it appears as if their analysis is demonstrating the ways in which cultural politics construct the clinical situation and the dynamics of patient care, Metzl and his colleagues fail to account for clinical attitudes toward sexuality as a significant vector of influence. In theorizing medical engagement with stigma and inequality in an article on structural competency in medical education, published in *Social Sciences and Medicine*, also in 2014, Metzl and Hansen argue that medical education needs to train health care professionals more systematically so that they can think about how such variables as race, social class, gender, and ethnicity shape and are shaped by the interactions between doctors and patients (Metzl and Hansen 2014, 127). It is no wonder that the development of structural competency in medical education seems to occlude sexuality, as it does so again here, given that 40% of physicians in the US reported in 2010 as to having no training in LGBT health in medical school or in their residencies (Fallin-Bennett 2015, 550). In addition, remnant homophobia in medical workplaces and schools, especially evident through homophobic remarks, has resulted in a reluctance for medical providers to come out. Fallin-Bennett cites a 2011 study by Mansh and White on the experiences of LGBT medical students, which was presented at the American Association of Medical Colleges annual conference; in the study, 16-17% of lesbians and gay men, 50% of bisexuals, and 60% of those who were transgendered did not disclose their sexual or gender identities in contexts related to medical school in the US (Mansh and White 2011; in Fallin-Bennett 2015, 550). In addition, Fallin-Bennett surmises, while acknowledging that this requires further study, that LGBT students may be more likely than their heterosexual or gender-conforming peers not to apply to medical school or to drop out once they are there (2015, 550).

With implicit and explicit bias toward them in medical and clinical settings, LGBT patients feel reluctant to come out to their medical providers for fear of discrimination and judgment given that these biases seem to be ignored, if not reinforced, in medical education and clinical training, which can negatively affect the quality of care received by LGBT patients. Historically, this may be because biomedicine has produced highly advanced knowledge of the biological impacts of lived environments with relatively undertheorized analyses of the environments themselves and their social and cultural impacts on medical decisions and patient care (Metzl and Hansen 2014, 129). Yet it is also historical to point out, as I have been arguing in this article, that healthcare systems and individual practitioners and researchers have systematically pathologized homosexuality and gender nonconformity, the latter most recently in children. As a result, LGBTQI patients have often undergone reparative conversion therapies, which have now been deemed inappropriate and harmful, and children born with DSD, or disorders in sex development, have been subjected to invasive and damaging interventions, including hormonal treatments and genital cosmetic surgery. More generally, and even without specific “corrective” treatments, the lingering, and still all-too-present actual, internalized, and anticipated medical and social stigmas experienced by LGBTQI people often result in risky behaviors amongst those in this vulnerable group that create significant disparities in their physical and mental health (Eckstrand and Sciolla 2014, 12, 14). It is important to note that the annual report of the United Nations Human Rights Council, published by the United Nations High Commissioner for Human Rights and entitled “Discrimination and violence against individuals based on their sexual orientation and gender identity,” stipulates quite clearly that conversion therapy and gender reassignment, when forced or involuntary, as well as unnecessary medical interventions involving intersex children, break the UN’s prohibition on torture (UN High Commissioner for Human Rights 2015, 11). Moreover, as I have been arguing, the Report also stipulates that discriminatory policies and practices of healthcare institutions adversely affect the quality of health services and deter patients from seeking them (UN High Commissioner for Human Rights 2015, 14). This implies radical analyses of new kinds of interventions, contestations, and struggles around the conflation of good health with conformity to gender and sexual norms, as well as further analysis into the contradictions between the urgency of ethical biomedical practice and critical healing alongside the various discourses, ideologies, and cultures which shape biomedicine, and by which biomedical knowledge and clinical practices are themselves shaped.
